# Effect of Trunk Sagittal Attitude on Shoulder, Thorax and Pelvis Three-Dimensional Kinematics in Able-Bodied Subjects during Gait

**DOI:** 10.1371/journal.pone.0077168

**Published:** 2013-10-29

**Authors:** Alberto Leardini, Lisa Berti, Mickaël Begon, Paul Allard

**Affiliations:** 1 MovementAnalysis Laboratory, Istituto Ortopedico Rizzoli, Bologna, Italy; 2 Laboratoire d'ingénierie du mouvement, Department of Kinesiology, University of Montreal, Montreal, Quebec, Canada; 3 Department of Kinesiology, University of Montreal, Montreal, Quebec, Canada; 4 Human Movement Laboratory, Research Centre, Sainte-Justine Hospital, Montreal, Quebec, Canada; University of Muenster, Germany

## Abstract

It has been shown that an original attitude in forward or backward inclination of the trunk is maintained at gait initiation and during locomotion, and that this affects lower limb loading patterns. However, no studies have shown the extent to which shoulder, thorax and pelvis three-dimensional kinematics are modified during gait due to this sagittal inclination attitude. Thirty young healthy volunteers were analyzed during level walking with video-based motion analysis. Reflecting markers were mounted on anatomical landmarks to form a two-marker shoulder line segment, and a four-marker thorax and pelvis segments. Absolute and relative spatial rotations were calculated, for a total of 11 degrees of freedom. The subjects were divided into two groups of 15 according to the median of mean thorax inclination angle over the gait cycle. Preliminary MANOVA analysis assessed whether gender was an independent variable. Then two-factor nested ANOVA was used to test the possible effect of thorax inclination on body segments, planes of motion and gait periods, separately. There was no significant difference in all anthropometric and spatio-temporal parameters between the two groups, except for subject mass. The three-dimensional kinematics of the thorax and pelvis were not affected by gender. Nested ANOVA revealed group effect in all segment rotations apart those at the pelvis, in the sagittal and frontal planes, and at the push-off. Attitudes in sagittal thorax inclination altered trunk segments kinematics during gait. Subjects with a backward thorax showed less thorax-to-pelvis motion, but more shoulder-to-thorax and thorax-to-laboratory motion, less motion in flexion/extension and in lateral bending, and also less motion during push-off. This contributes to the understanding of forward propulsion and sideways load transfer mechanisms, fundamental for the maintenance of balance and the risk of falling.

## Introduction

The trunk plays an important role in human locomotion. It accounts for more than 50% of the body weight and its kinematics has been associated with age-related changes [1] and with maintenance of dynamic stability in elderly individuals [2]. Trunk motion is therefore of large clinical interest, not only in the presence of spinal pathologies [3] but also in patients with Parkinson [4] or cerebral palsy [5] diseases. Furthermore it was reported that sagittal and frontal variability in trunk accelerations could be indicative of balance dysfunction in elderly individuals [6]. In particular, the natural attitude of trunk inclination seems to affect able-bodied gait [7]–[9], and it was demonstrated that an original forward (FW) or backward (BW) inclination is maintained at gait initiation [10] and during locomotion [11]. It also appeared that women maintain a greater trunk extension than men [12]. Despite these evidences, no studies have shown the extent to which shoulder, thorax and pelvis kinematics are modified during gait by this FW or BW inclination attitude. This information could prove to be of importance since older people modify their gait patterns to ensure that head and pelvis remain stable [13].

In this respect, classes of gait patterns were identified in able young adults [14] and in physically active men over 70 years of age [15]. These classifications, however, were sought as based on lower limb dynamics and did not consider the effect of trunk inclinations. In a more recent study [11], two different gait patterns were found associated with FW and BW inclination attitudes of the trunk. In particular, hips and thoracolumbar spine net muscular extension moments were higher for the FW than for the BW group, indicating that other body segments are perturbed by trunk natural inclination. Pelvis and lumbar spine general motion in walking is well documented in the literature [16]–[18], and thorax motion alone was also tracked to assess the gender effect in normal adult gait [12], but kinematics interactions between the thorax and the other adjacent body segments have not been established.

This lack of information can also be accounted for by the currently available trunk kinematics models, which vary much in complexity and in the number of skin markers but employ mostly only a single rigid segment. From a recent comparative study of eight such models for clinical gait analysis [19], it can be concluded that the larger is the number of markers and segments, i.e. covering the shoulders, the thorax, the lumbar spine area and the pelvis, the more complete is the description of this complex kinematics during gait. The effect of thorax inclination on the adjoining body segments can be determined potentially by means of the most complete of these models, possibly to point out likely compensating effects in other segments and in other anatomical planes.

The coupling of trunk motion in the three anatomical planes during gait has intrigued a number of researchers. Whittle and Levine [17] reported a strong relationship between lumbar lordosis and pelvic tilt, and also between trunk bending and pelvic obliquity, even though this varied between subjects. Chung et al. [12] showed that thorax and pelvis motion is coupled in the frontal and the transverse planes, whereas sagittal plane motion is mainly to counterbalance the asymmetric kinematics of the lower limbs. Leardini et al. [20] reported motion at the pelvis, thorax, shoulders and spine for a large number of elementary exercises and activities of daily living. Consistent patterns were observed for most kinematic measurements, both intra- and inter-subject, which also revealed large coupling between rotations in all three anatomical planes. The results from these studies support also the hypothesis of subject specific trunk sagittal inclinations, and relevant possibly important effects on trunk segments motion on the other two anatomical planes.

The effects of trunk inclination on lower limb joint kinematics [9] and on joint moments have been described in healthy subjects [21] and in patients with osteoarthritis [22]. However kinematics changes in the upper and lower trunk during gait due to its natural inclination in the sagittal plane remain to be demonstrated. Improper sensing of the trunk spatial orientation in elderly individuals could result in incorrect foot positioning during gait, which could lead to insufficient frontal plane stability and higher risk of falling [2].

The general objectives of this study are to test the effect of FW and BW thorax inclinations on pelvis and upper trunk segment kinematics during gait. In particular we want to determine, together with possible gender-based differences, a) which inter-segmental motion (Segments) and b) which anatomical axis rotation (Axes) are affected the most, and in case c) the gait cycle periods when these kinematics perturbations occur (Periods). A group of able-bodied persons was analyzed to address these objectives.

## Materials and Methods

### Ethics statement

The data here analyzed are gathered from a series of internal tasks and projects of the gait analysis laboratory, all approved by the institutional scientific review board (Istituto Ortopedico Rizzoli, Bologna, Italy). In any case, an informed consent was signed by each participant volunteering for the study, after explanation of the possible benefits; no risks are associated to the present experiments of gait analysis by optical stereophotogrammetry.

### Subjects and data collection

Thirty young subjects volunteered for the study (Table 1) and were analyzed with video-based motion analysis. Marker motion was recorded with an eight-camera motion capture system (Vicon 612, Vicon Motion Systems Ltd, Oxford, UK), at a sampling rate of 100 Hz. To label the markers, reconstruct, and in case interpolate the three-dimensional trajectories, Vicon standard software tools were used. In particular, marker trajectories were filtered using Woltring's smoothing splines [23], implemented in these tools. Three trials of level walking were collected for each subject. Gait cycle phase identification was obtained by a single experienced operator, based on force plates and foot marker trajectories. In particular the initial heel strike was identified at the first not null signal for the ground reaction force, the final when the kinematics variables showed similar events within the complete patterns. The selected data intervals were resampled on a 0–100 basis to allow for statistical analysis.

**Table 1:** Anthropometry and thorax inclination.

N°	FW/BW	Gender	Age (years)	Mass (kg)	Height (cm)	Thorax inclination angle (degree)
1	BW	F	27	60	181	−0.3
2	BW	F	21	46	158	2.4
3	BW	F	27	50	170	3,3
4	BW	F	26	54	168	7.9
5	BW	F	31	49	173	7.9
6	BW	F	24	50	165	8.9
7	BW	F	25	48	156	9.0
8	BW	F	27	54	167	9.5
9	BW	M	26	73	179	9.8
10	BW	M	24	55	168	11.1
11	BW	M	28	80	180	11.2
12	BW	M	26	68	178	11.7
13	BW	F	28	54	167	11.7
14	BW	M	19	78	180	12.4
15	BW	F	25	55	164	12.4
**Avg**			**25.6**	**58.3**	**170.3**	**8.6**
**St.dev.**			**2.9**	**11.1**	**8.0**	**3.9**
16	FW	F	28	52	168	13.2
17	FW	M	30	69	167	13.4
18	FW	F	28	52	163	13.5
19	FW	M	22	75	173	14.1
20	FW	M	25	80	183	14.8
21	FW	M	26	85	183	15.1
22	FW	M	31	106	190	15.5
23	FW	F	25	62	163	15.5
24	FW	F	26	48	160	15.7
25	FW	M	24	69	178	16.0
26	FW	M	29	99	192	18.0
27	FW	M	39	78	180	18.2
28	FW	M	24	69	178	18.4
29	FW	M	24	75	175	18.9
30	FW	F	28	59	172	21.3
**Avg**			**27.3**	**71.9**	**175.0**	**16.1**
**St.dev.**			**4.1**	**16.6**	**9.7**	**2.4**

Anthropometric data of the population analyzed, divided already in those with FW or BW thorax inclination. The mean thorax inclination angle over the three repetitions of level walking are also reported in the last column (increasing order). For each group, corresponding averages (Avg) and standard deviations (St.dev.) are reported.

### Gait analysis protocol

**Figure 1:**
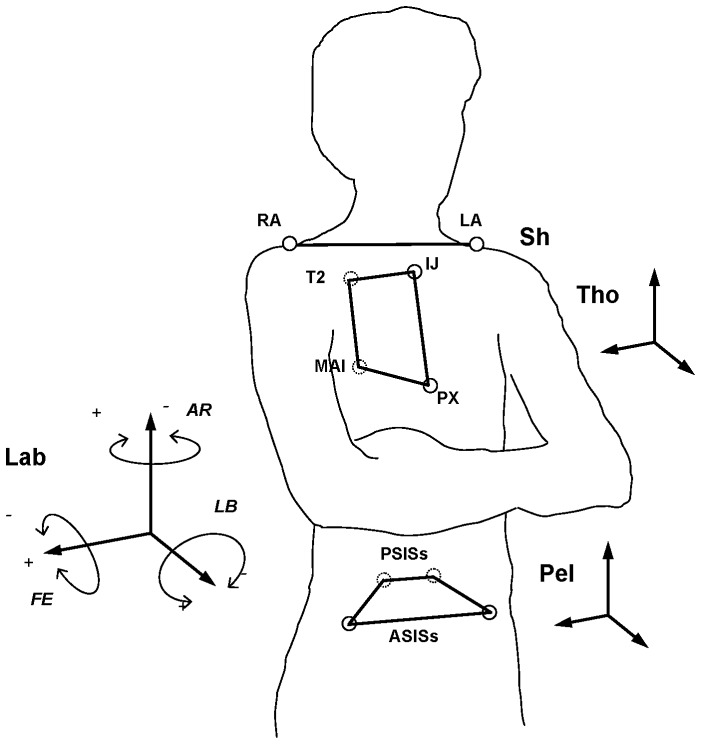
Diagram of the conventions. Diagrammatic representation of the body segments analyzed with relevant skin markers, together with the convention for the three relevant rotations [20]. At the pelvis, the right and left anterior superior (ASIS) and posterior superior (PSIS) iliac spines were tracked. At the trunk, the deepest point of incisurajugularis (IJ), i.e. the suprasternal notch, the xiphoid process, i.e. the most caudal point of the sternum (PX), the spinous process of the second thoracic vertebrae (T2), the midpoint between the inferior angles of most caudal points of the two scapulae (MAI), and the right and left acromions (RA, LA)were tracked.

Multi-segmental trunk motion was analyzed during level walking, according to an established protocol [20]. This implies tenspherical markers, 14 mm in diameter, glued directly onto the skin with bi-adhesive tape in correspondence with anatomical landmarks (Figure 1). At the pelvis, the anatomical reference frame (Pel) was defined by a medio-lateral axis between the right and left ASIS (positive to the right), and by a vertical axis orthogonal to the transverse plane, through the ASIS and the mid-point between the two PSIS. A thorax segment (Tho) was tracked by the optimal spatial matching of four thoracic markers, T2, MAI, PX and IJ, with a vertical axis between MAI and T2, and a medio-lateral axis orthogonal to the sagittal plane defined by PX, MAI and T2. The antero-posterior axis is orthogonal to the other two, and forms the thorax transverse plane together with themedio-lateral axis; the frontal plane is formed by the medio-lateral and vertical axes. A separate shoulder line segment (Sh) was defined between the two acromion markers, RA and LA. Three-dimensionalabsolute rotations of the thoracic (Tho/Lab) and pelvic (Pel/Lab) anatomical frames with respect to the global laboratory frame, together with relative rotations between the pelvic and the thoracic frames (Tho/Pel), were calculated in accordance with the standard joint convention [24], thus obtaining flexion/extension (FE), lateral bending (LB) and axial rotation (AR) in the sagittal, frontal and transverse planes respectively (Figure 1). Planar rotations of the shoulder line segment (Sh) in the frontal and transverse planes of the thorax segment (Sh/Tho) were also calculated.

### Statistical analysis

Out of these 38 dependent variables, 16 had a Shapiro Wilk's W test that displayed a normal frequency distribution. For the others their corresponding p values were as follow: 7 with a p less than 0.1, 7 variables with a p greater than 0.1 but less than 0.2, and 8 variables with a p larger than 0.2.

The mean thorax sagittal inclination in the laboratory frame was calculated over the gait cycle and averaged over the three trials for each subject (Table 1). Afterwards the individual average thorax inclination values were arbitrarily divided using the median as the division point, as in previous studies [25], [26]. Because our subjects formed mixed gender groups, a multivariate analysis (MANOVA) was performed to test its effect on all the shoulder, thorax and pelvis kinematics parameters. These parameters were the peak values (minima and maxima) of the angle time-histories occurring during the gait cycle of the right dominant lower limb. If a gender effect was present then height and mass would be included as co-variables in the subsequent analyses to account for morphological differences. The level of significance was set a priori at α = 0.05 for all analyses by the Wilk's Lambda test.

A nested design ANOVA is carried out when the objectives are not to compare every level of a factor (A) with every level of another factor (B). Thus, to test the effects of FW and BW thorax inclination (Factor A) on three separate independent variables (Factor B), namely, body Segments (Sh/Tho, Tho/Lab, Tho/Pel and Pel/Lab), Axes of rotation (FE, LB and AR) and gait Periods (Heel-strike, Mid-stance, Push-off and Swing) two-factor nested design ANOVAs were performed. In other words we wanted to determine if the variability was due to the difference between the thorax inclination groups (Factor A) or because of the variability among each Factor B (a-Segments, b-Axes or c-Periods). If a significant difference (p<0.05) was found then planned comparison was made with the Bonferroni adjustment for the pairwise comparisons to determine where the differences occurred. Statistica (version 6, Chicago, Ill.) was used for all statistical analyses.

## Results

**Figure 2:**
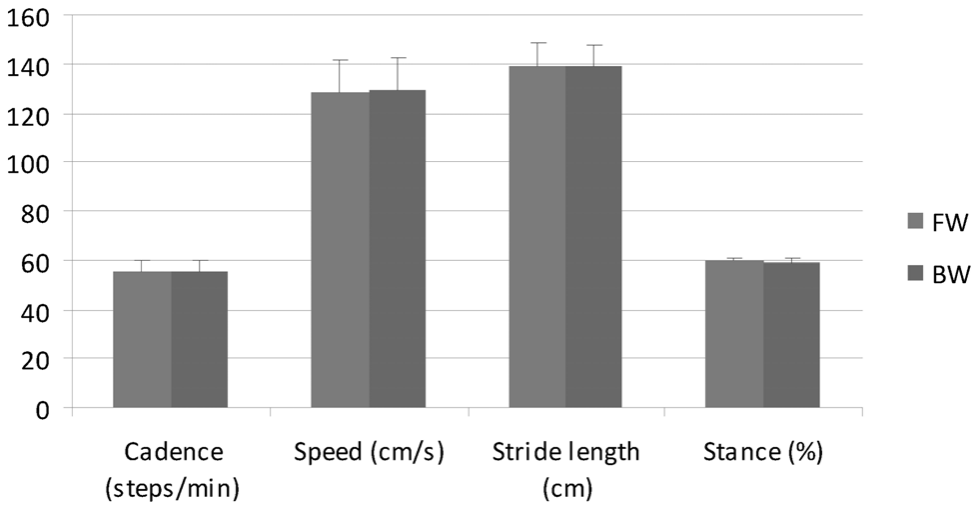
Spatio-temporal parameters. Spatio-temporal parameters for the FW and BW groups; means and standard deviations.

**Figure 3:**
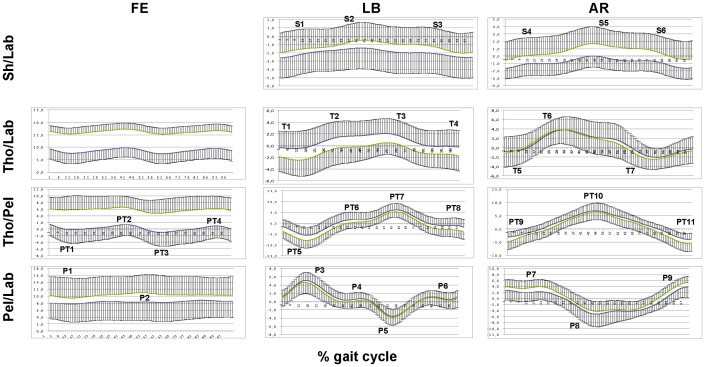
Patterns of rotation. Sh/Tho, Tho/Lab, Tho/Pel, and Pel/Lab motion (four rows) about the three anatomical axes (three columns) is reported in terms of the average and one standard deviation (in only one direction), for both FW (green) and BW (blue) inclination groups, over the gait cycle. Relevant maximum-minimum peaks are also depicted by corresponding alphanumeric codes. Unit is degree.

There were small differences in the anthropometric and temporal gait parameters between the FW and BW groups (Table 1, Figure 2), and only mass was statistically larger (p = 0.0134) in the FW group, by 13.6 kg on average. This could be attributed to the greater number of male subjects in this group. Consistent patterns of motion were observed within the two thorax inclination groups (Figure 3). In a number of plots, and in particular in certain gait periods, these patterns were clearly distinct. The results of the MANOVA showed group (p = 0.0246) but no gender (p = 0.8209) effects, and no interaction (p = 0.6625).

**Figure 4:**
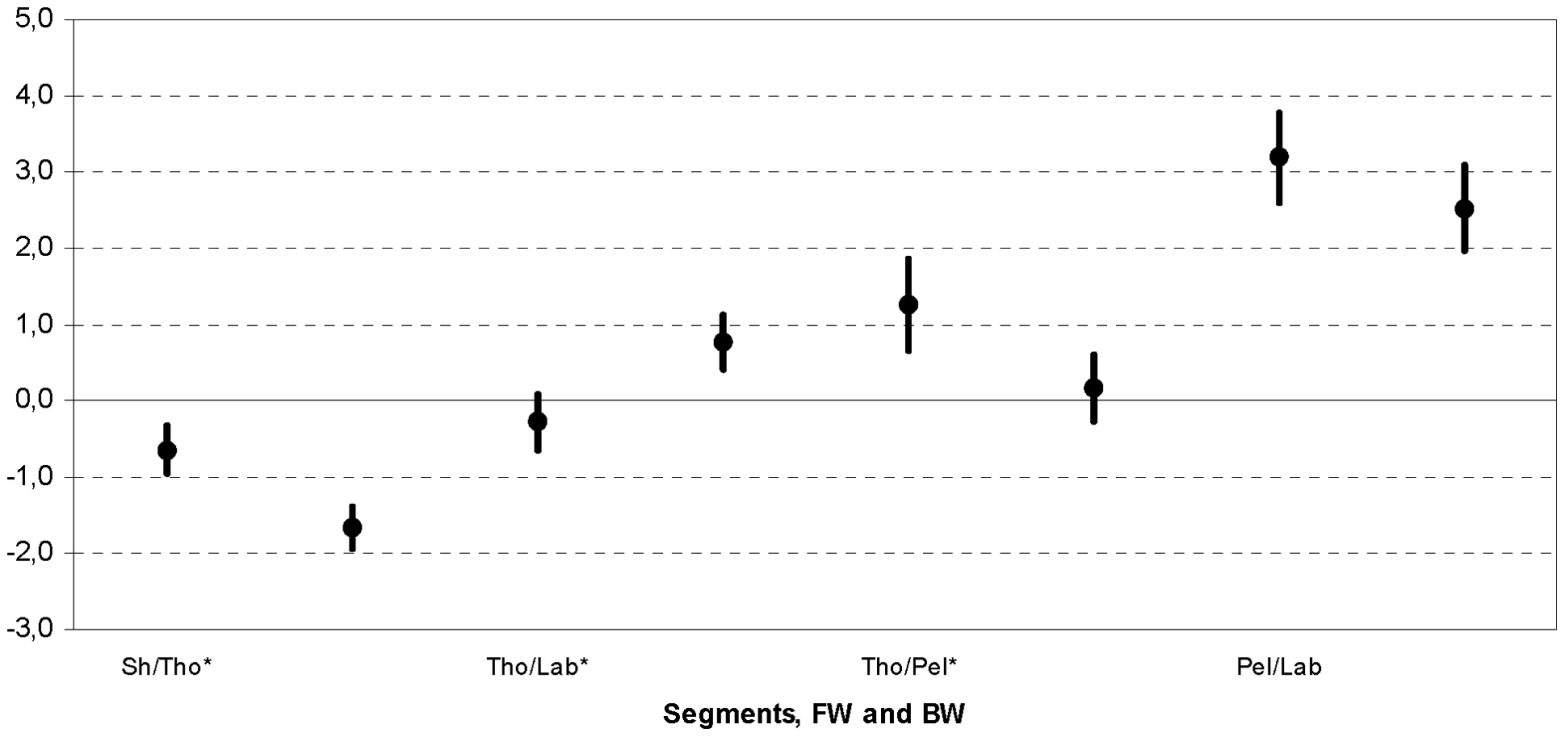
Nested ANOVA for a-Segments. Nested ANOVA: 95% confidential interval for body Segments; for each (Sh/Tho, Tho/Lab, Tho/Pel, Pel/Lab), group FW and BW are reported. Those with significant difference are denoted with *.

**Figure 5:**
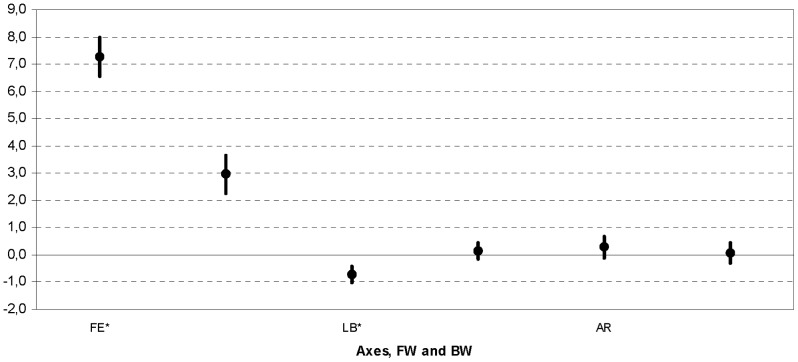
Nested ANOVA for b-Axes. Nested ANOVA: 95% confidential interval for rotation Axes; for each (FE, LB, and AR), group FW and BW are reported. Those with significant difference are denoted with *.

**Figure 6:**
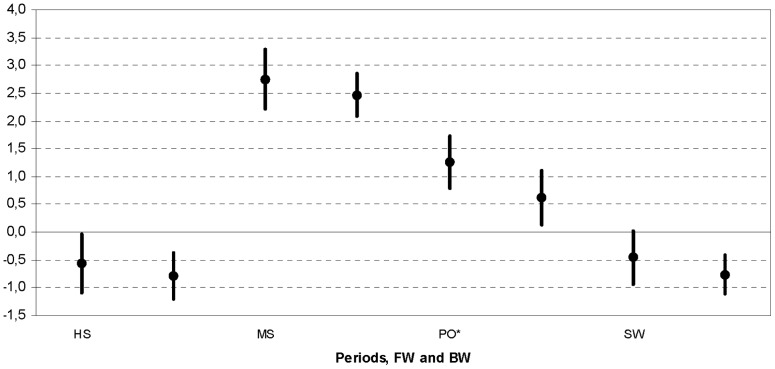
Nested ANOVA for c-Periods. Nested ANOVA: 95% confidential interval for rotation Axes; for each (HS  =  heel-strike, MD  =  midstance, PO  =  push-off and SW  =  swing), group FW and BW are reported. Those with significant difference are denoted with *.

a) All the nested ANOVA revealed Groups effect denoting that subjects with a FW or BW thorax inclination behaved differently and that depended on the second factor. First of all, a statistical difference (p = 0.0193) was found for Segments (Figure 4). Subsequent statistical analysis denoted that BW subjects performed more motion at the Sh/Tho (p = 0.0170) and Tho/Lab (p = 0.0047) but less at the Tho/Pel (p = 0.0006). Pel/Lab motion was similar for both groups (p = 0.0575).

b) The nested ANOVA for Groups and Axes (Figure 5) showed a statistical difference (p<0.000). Further statistical analyses revealed that there was a significant difference in FE (p<0.000) and in LB (p = 0.0007), but not in AR (p = 0.4323). Motion magnitude was always smaller for BW.

c) The nested ANOVA for Groups and gait Periods (Figure 6) also showed a statistical difference (p<0.000). Both groups behaved similarly at heel-strike (p = 0.4057), during midstance (p = 0.5090) and swing (p = 0.3329). Only push-off values were significantly smaller (p = 0.0464) in subjects with BW inclination.

## Discussion

Trunk sagittal inclination is known to perturb lower limb gait patterns in both able-bodied subjects [11] and in people with physical disabilities [3]. In particular, the trunk counterbalances the lower limbs forward advance and reduces body rotations prior to heel-strike [12]. Modifying the distribution of the masses in the trunk also affects gait patterns in scoliotic adolescents [27]. Inversely, it can be reasonably assumed that changes in trunk inclination would perturb its kinematics. To our knowledge no study has yet reported three-dimensional trunk segment kinematic patterns from subjects with different sagittal inclination attitudes. To explore on motion between distinct segments, a trunk model that includes more than a single rigid body was exploited [20], for which feasibility and intra-subject repeatability had been shown [20]. Markers and reference frames were embedded on the pelvis, thorax and shoulders for these three segments to be tracked separately. In particular, for the thorax segment, the optimal three-dimensional position and orientation in the laboratory frame was estimated by four largely distributed and well identifiable landmarks, which describes better its overall rigid motion also with respect to adjoining body segments, i.e. shoulders above and pelvis below. The extent to which these three segments, assumed to be rigid, contribute individually to stability and progression during locomotion has not been established yet.

Sagittal thorax inclination was found to modify motion in upper trunk segments but not in the pelvis. Generally the mobility of Sh/Tho and Tho/Lab segments was more pronounced with BW thorax attitude, but was also associated with reduced Tho/Pel mobility (Figure 4). This smaller lower back mobility could be representative of a straighter back or a position closer to the vertical thus requiring smaller lumbar extension moments. This near vertical attitude could be a means to reduce axial loading to the spine especially in low back pain where abnormal trunk postures in the sagittal plane [28] and limitations in the maximal range of lumbar motion [29] were reported. Subjects with a natural FW thorax inclination were found to develop higher lumbar extension moments [11]. This was also observed in individuals where FW thorax inclination is induced by backpack loads [30]. These muscle extension moments could be explained by an anterior displacement of the trunk's center of mass, as well as attributed to a greater muscular capability to control the upper body during gait.

Trunk segments kinematics was previously reported during gait of able-bodied subjects [10], [19], [20] but only a few addressed its coupling motion. Synchronization or coupling between the upper trunk and the pelvis has also been discussed [17], [20]. Trunk sagittal motion occurs to counterbalance the cyclic motion of the lower limb during the swing phase and to reduce the angular velocity toward the contralateral side in the frontal plane [12]. Our study is the first to report the effect of trunk sagittal inclination on motions in all three anatomical planes of the shoulder, thorax and pelvis in the laboratory frame, together with their relative motion. Subjects with a BW inclination showed less coupling in flexion/extension and lateral bending (Figure 5). This is in agreement with reduced thorax mobility observed in this group. Apparently, with a straighter trunk attitude during gait the compensatory movements which occur in other planes are also reduced. Differences in spatio-temporal parameters between the two thorax inclination groups were expected in this study. We did not find significant differences in these variables, and this can be accounted for by the spinal movement compensations which are linked to the motions of the lower limbs during walking [31].

Moreover, possible different effects of thorax sagittal inclination along the gait cycle were investigated, in particular by looking at four adjacent periods, i.e. heel-strike, midstance, push-off and swing (Figure 6). This division was based on the observed occurrences of peaks along the measured rotations (see Figure 3). Individuals with thorax BW inclination showed less mobility than those with FW inclination, but only during push-off. This is of clinical relevance since weight transfer from the supporting to the contralateral limbs occurs exactly during that period. A substantial amount of mechanical work is required in the frontal plane and particularly at push-off to control the pelvis and trunk motions [32]. In patients with hip osteoarthritis or after arthroplasty, this could be an important issue, because what it is usually described as a Trendelenburg in reality is a weak hip abductor effect. It is important to point out that the present analyses tested also whether gender does play a role in these results; it was demonstrated that this factor is not a relevant co-variable.

The results of this study could justify in part the reason why elderly people have a tendency to lean forward during gait [1]. This could be explained by the need to maintain dynamic stability [2] and to reduce possibly the risk of falling. Push-off of the supporting limb occurs when the contralateral limb makes initial foot contact during double support. Incorrect foot positioning could increase the risk of falling [2]. Trunk spatial orientation control and increase lower back motion by leaning forward could improve lateral balance in elderly individuals [33]. These results support the potential of using gait retraining for walking with forward trunk inclination in the elderly with a straighter back or people with poor balance control and risk of falling.

## Conclusion

Sagittal thorax inclination attitude altered three-dimensional kinematic patterns of the upper trunk segments during natural gait. As for the original three objectives, (a) subjects with a backward thorax inclination showed less thorax-to-pelvis motion, but more shoulder-to-thorax and thorax-to-laboratory motion. Overall, (b) these subjects also showed less motion in flexion/extension and in lateral bending. This could be a mechanism to reduce axial loading on the lumbar spine. (c)Subjects with a backward thorax inclination also displayed less motion during push-off, when perhaps the body is both propelled forward and sideways to transfer from one supporting limb to the other. This could be critical in the maintenance of balance especially in the elderly or people with poor balance control and risk of falling, and therefore to be investigated in the future. Finally, the present investigation also demonstrated that gender does not affect these results and conclusions.
